# Household electricity access in Africa (2000–2013): Closing information gaps with model-based geostatistics

**DOI:** 10.1371/journal.pone.0214635

**Published:** 2019-05-01

**Authors:** Ricardo Andrade-Pacheco, David J. Savory, Alemayehu Midekisa, Peter W. Gething, Hugh J. W. Sturrock, Adam Bennett

**Affiliations:** 1 Malaria Elimination Initiative, Institute for Global Health Sciences, UCSF, San Francisco, CA, United States of America; 2 Big Data Institute, Nuffield Department of Medicine, University of Oxford, Oxford, United Kingdom; Syracuse University, UNITED STATES

## Abstract

Household electricity access data in Africa are scarce, particularly at the subnational level. We followed a model-based Geostatistics approach to produce maps of electricity access between 2000 and 2013 at a 5 km resolution. We collated data from 69 nationally representative household surveys conducted in Africa and incorporated nighttime lights imagery as well as land use and land cover data to produce maps of electricity access between 2000 and 2013. The information produced here can be an aid for understanding of how electricity access has changed in the region during this 14 year period. The resolution and the continental scale makes it possible to combine these data with other sources in applications in the socio-economic field, both at a local or regional level.

## Introduction

Household-level access to electricity is strongly associated with socioeconomic status and an important indicator of economic development [1]. Improving access to electricity is a global challenge that if addressed, can help to reduce poverty and inequalities in health outcomes and education attainment [2–4]. Understanding and tracking rates of electricity access within and across countries offers opportunities for monitoring patterns of development, and determining areas of unmet need.

Countries with strong National Statistical Systems (ensembles of statistical organizations that produce information on behalf of the government) are able to produce regular and timely information on electricity access, as well as other development indicators, such as health. In some cases, the information production of such systems has been standardized and integrated between countries [5, 6]. These efforts are particularly important when dealing with matters that occur beyond the borders of a single country, like global health, natural hazards or international commerce [7–10]. However, strong and integrated Statistical Systems are not prevalent in developing regions such as sub-Saharan Africa (SSA) [11, 12]. Often data are only available at country level, masking important within country heterogeneity.

In recent years, international efforts have improved the tracking of development and population health in low income countries through the implementation of large nationally representative household surveys [13]. For example, the Demographic and Health Surveys (DHS) are intended to assist participant countries in the collection and use of data to monitor and evaluate population and health. DHS has been active for many years and is one of the main references for metrics of sociodemographic changes, including electricity access [14]. However, due to the varying periodicity and spatial coverage of these surveys, the information they generate presents gaps in space and time.

The implementation of regular and representative surveys is an expensive task. Nevertheless, today’s greater availability of remote sensing data allows an opportunity to indirectly measure household access to electricity [9, 15–20]. For example, the National Oceanic and Atmospheric Administration (NOAA) have been maintaining global nighttime light (NTL) imagery produced by US Air Force satellites since 1992. Such images provide repeatable data at global scales. Nighttime light images do not immediately translate into electricity access of households. Yet, the information they provide can be calibrated with available data on this topic; in particular, DHS data.

Previous studies have examined population density, urban extent, or produced poverty maps using satellite nighttime lights data [21–23]. Here we describe the use of household survey data, satellite imagery, land-cover data and geostatistics modeling to construct a time series of electricity access across Africa from 2000 to 2013. The precision score of our model is 85.70%. We were motivated to produce these layers as part of work exploring changes in malaria incidence due to anthropogenic dynamics across Africa. We found a missing gap of data at subnational level in this topic, and we decided to share these information products with the methodology used as we think other professionals working in related fields could also benefit from them.

## Data sources

### Household survey data

The DHS program has developed standard procedures and methodologies to collect and disseminate representative household-based data of participating countries on topics related to population and health [24]. Annual indicators of electricity access per country, among others, are available through the DHS Stat Compiler (URL in Section A in S1 File). GPS sample locations of the surveys can also be accessed upon request (URL in Section A in S1 File). A majority of low to middle income countries, including 44 African countries, globally participate in this program. This provides coverage of most of the continent, although data are not available for every year.

Other valuable sources of information are the Malaria Indicator Surveys (MIS) and the AIDS Indicator Surveys (AIS). MIS were developed to support the global fight against malaria by the Roll Back Malaria partnership [25]. This program covers 25 countries worldwide and collects data with a frequency ranging between 2 and 5 years. Similarly, AIS were developed as a standardized tool for monitoring national HIV/AIDS programs [26]. Although the main concern of MIS and AIS is to collect data related to prevention and morbidity of these diseases, they also collect information on electricity access, among other household-level statistics. To the authors knowledge, DHS, MIS and AIS follow the same methodology and their information is comparable.

**Fig 1:**
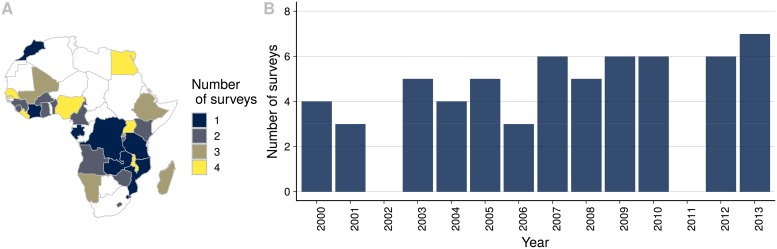
Data sources. Data available per country and year. A: Number of surveys conducted per country. B: Number of surveys conducted per year.

In total we used sample points from 69 different surveys including DHS, MIS and AIS (Section B in S1 File contains a summary of the surveys used in this study). During the 14 year period that this study covers, 2000-2013, we found that no country had more than 5 surveys conducted. Fig 1A shows a map with the number of surveys conducted per country and Fig 1B shows the number of surveys conducted per year. This information included the number of households that have access to electricity and geolocation. Part of the methodology of these surveys consist of protecting the privacy of the respondents: first, by aggregating households into clusters and only reporting data at cluster-level, and second, by randomly displacing the cluster centroid (geolocation reported). Rural points are randomly offset up to 5 km, with 1% moved up to 10 km; and urban points are moved up to 2 km.

### Nighttime lights

NOAA has provided access to annual composite NTL images since 1992. Satellite images of nocturnal lighting are indicators of human activity at both global and local scales. Although the frequency and spatial resolution of these images goes beyond any survey capabilities, they have inherent limitations. Images are not calibrated between years nor between satellites. Using raw data can be therefore misleading. NTL images have to be denoised and processed to be comparable across time [27]. The analysis presented here uses a series of images from 2000 to 2013 already calibrated (URL in Section A in S1 File). They consist of a series of raster files representing the level of luminosity with a resolution of approximately 1 km. The intensity of light is encoded in a 6-bit dynamic range with values between 0 and 63 (0 meaning no light captured). The authors of these images have also proved that they are highly correlated with gross domestic product and urbanization [28].

### Land use and land cover

Another data source that can help inform sociodemographic changes across space is Land Use and Land Cover (LULC) [29, 30]. We used a series of LULC data from 2000 to 2013 (URL in Section A in S1 File). This data consist of raster layers where pixels are classified into 7 categories: impervious surface, low biomass, high biomass, bare soil, sand, rock and water. The authors of these data quantified land cover and impervious surface changes over 16 years in Continental Africa and Madagascar using a pixel resolution of 30 m [31]. It is worth mentioning that NTL data is somehow already embedded in the LULC data; the authors of these dataset used the calibrated NTL images introduced above as an input. Impervious surfaces include: asphalt roads, concrete, metal roofs and other built infrastructure. In general, we can understand impervious surfaces as man-made changes in the natural land cover related to urbanization. Low biomass includes crop fields, grass and shrubland; and high biomass consists of dense forest.

**Fig 2:**
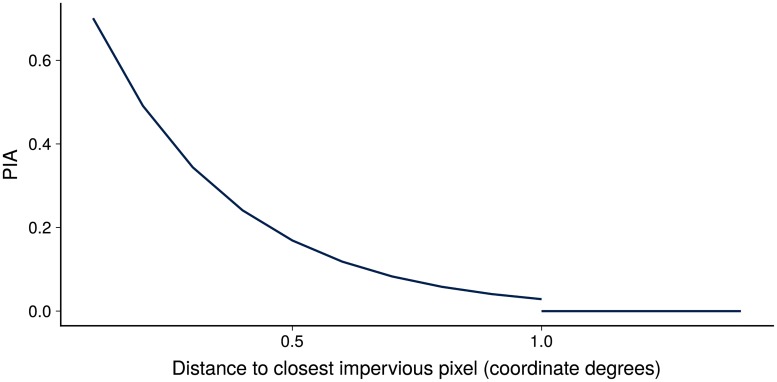
Proximity to impervious area. Decay function transformation applied to the closest distance to an impervious pixel. For distances larger than 1, PIA is defined as zero.

In addition to the LULC categories, we constructed two additional variables: impervious area proportion (IAP) and proximity to an impervious area (PIA). The high resolution of the LULC data was un-necessary in our application, given that we worked with a 5 km resolution. Hence, we downscaled the LULC layers to 5 km. Nevertheless, as a means to encode the high resolution data on impervious surfaces (which is related to urbanization) we computed the variable IAP. This is the proportion of high resolution pixels classified as impervious within the 5 km pixel. Another variable that we considered important was the distance to *urban centers*. We defined this variable as the Euclidean distance to the closest pixel defined as impervious. To speed up computations we limited the search for the closest impervious pixel to a radius of 1 degree. Then to ease imputing data at the locations beyond the 1 degree radius we re-expressed the distance with a decay function (bounded from below at zero) as

$$\begin{array}{c} \text{PIA}\left(\text{x}\right)=\text{exp}(-\frac{{\mathrm{argmin}}_{{\text{x}}_{u}}∥\text{x}-{\text{x}}_{u}∥}{\sigma_{u}}), \end{array}$$ (1)

where **x**_*u*_ is a pixel classified as impervious, and *σ*_*u*_ is the standard deviation of ${\text{argmin}}_{{\text{x}}_{u}}∥\text{x}-{\text{x}}_{u}∥$ for all locations in the survey. In this case *σ*_*u*_ = 0.28. For this new variable we assigned a value of zero to all pixels with missing value. Fig 2 shows the shape of the decay function used. After the value of 1, all values are defined as zero.

### Population

To analyze and discuss our results we used population estimates from the Gridded Population of the World (GPW) Series of the Socioeconomic Data and Applications Center. The GPW project provides population estimates on a gridded surface defined across the globe [32]. This product is available in the form of raster files of approximately 1 km resolution for the years 2000, 2005, 2010 and 2015 (URL in Section A in S1 File). For the years within quinquennials, the population of each cell in the grid was estimated assuming a geometric growth.

## Methods

Our study covers only Africa’s mainland (48 countries) and Madagascar. Smaller islands and archipelagos (São Tomé and Príncipe, Seychelles, Comoros, Mauritus and Cape Verde) were not analysed. We sought to determine sub-national probabilities of household electrification for the period 2000-2013. We did this by studying the relation of this variable with NTL and LULC data through a geostatistic model. Since the size of the data used (30,115 data points from the 69 surveys) and the number of the predicted points (around 1.5 million per year, from 2000 to 2013) turns out to be limiting for doing exact inference, we relied on the Integrated Nested Laplace Approximation (INLA) [33, 34]. The technical details of this model are described in Sections C and D in S1 File.

As it was mentioned above, most of the survey data has been obfuscated by aggregating the household locations into clusters and displacing their location. This is a source of noise that will affect any statistical inference done on these data. As a means to reduce the effect of these noise source, DHS suggests to compute covariate values by averaging across a 5 km buffer around the GPS location reported [35]. We followed the approach suggested and kept our results at a 5 km resolution. In addition, we performed a sensitivity analysis to asses the impact of the locations displacement. We used a synthetic dataset where we displaced the locations, according to DHS methodology. The details of such analysis can be found in Section E in S1 File. As it is expected, a random displacement of pixel locations modify the parameter estimates (fixed effects parameters and random effects hyperparameters). However, our results show that these estimates are still consistent with the ones obtained from data that has not been displaced and also with the ground truth parameters. We also compared the sum of squared errors (SSE) of the predicted target values vs the ground truth. We observed that the displacement of locations resulted in an increment of 5.52% in the SSE when compared to the predictions using non-displaced data.

The model used was chosen among four candidates. All these models included as fixed effects NTL and the LULC related variables (the category and the two constructed variables). The first model was as a GLM with the fixed effects only; the second model included a spatial random effect; the third model included a spatial random effect too, but also included year as a fixed effect; the fourth model included a spatiotemoral random effect instead of both the spatial random effect and temporal fixed effect. We selected model 3 as the most adequate according to the conditional predictive ordinate (CPO). The details of the model selection process and a comparison of CPO values are presented in Section F in S1 File.

During the model selection phase we only used 70% of the survey points with displaced locations (21,080 observations). The remaining observations were used to assess the overall performance of model 3. As validation set we used the remaining 30% of the observations (9,035 data points). Table 1 shows the composition of both sets used along the analysis. Note that observations in these sets correspond to clusters of households. On average clusters have 24 households, however their number varies largely and they can contain from 1 to over 100 households.

**Table 1:** Datasets composition. Dataset partition used in the analysis. Set T was used for training and model selection, V1 was used for validation.

Set	Observations	Unit Reported	Displaced	Electricity
T	21,080	Cluster	Yes	34.64%
V1	9,035	Cluster	Yes	34.23%

We predicted the probability of a household having electricity at each of the locations in V1 and classified them into two groups: *with electricity* if the probability estimate was above 0.5 and *without electricity* otherwise. To test the performance of our model we randomly generated class instances according to the observed proportions of electricity access per cluster. Section G in S1 File contains more details of the procedure followed to generate such instances. The validation metrics computed will be discussed in the next Section.

## Results

**Fig 3:**
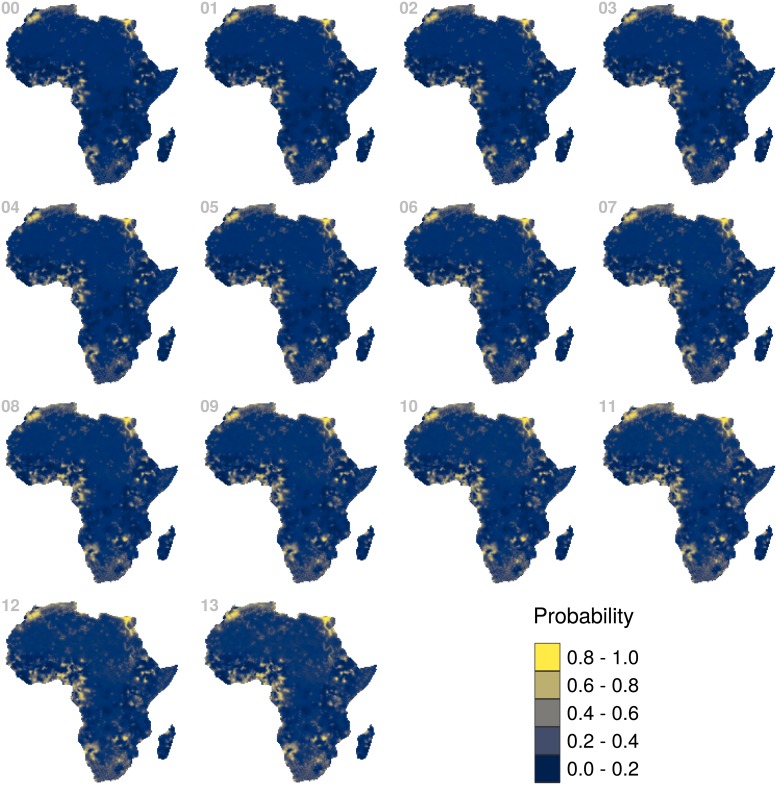
Probability of electricity access. From top left to bottom right the panels show the probability estimates for the years 2000 to 2013.

For each year in the analysis (2000-2013) we estimated the probability of electricity access in a 5 km grid across mainland Africa and Madagascar. In Fig 3 we show a timeline of such probabilities. Across these maps we can see how the central region of the continent has been historically less likely to have access to electricity. Nevertheless, the trend shows that the continent has been lighting up; either because the spots with electricity have been expanding or because new clusters of light have been popping up.

To assess the model’s performance, we predicted the synthetic classes of set V1. Table 2 summarizes the results using as classification threshold a predictive probability of 0.5. We classified correctly 85.70% of the cases in set V1. To better understand the performance of our model, beyond the number of corrected classified examples we computed the precision (or positive predictive value) and the sensitivity (or recall) of each test set. These results are presented in Table 3. The precision represents the proportion of locations predicted as *with electricity* that were correct, and the sensitivity represents the proportion of locations observed as *with electricity* that were correct. When predicting a location with electricity access, the model was right in 76.59% of the cases. Also the model was able to identify 84.82% of the locations with electricity.

**Table 2:** Confusion matrix of set V1. Percentages computed with the synthetic classes generated per cluster.

		Actual class	
		No electricity	Electricity
Predicted class	No electricity	56.18%	5.28%
	Electricity	9.02%	29.52%

**Table 3:** Validation metrics. Metrics computed on set V1.

Metric	Score
Accuracy	85.70%
Precision	76.59%
Sensitivity	84.82%

## Discussion

In this work we defined a model to translate imagery data into a probability of electricity access. The period of study (2000-2013) was constrained by the availability of inputs in a standardized format: NTL and LULC. Recent work has been done on NTL imagery to provide access to high quality and more recent data. For example, the Black Marble NTL product suit [36] contains information from 2012. However, such data sources are not necessarily comparable across time with the datasets we are using. In the case of the Black Marble suit, combining their data with NTL data used here would require the development of a calibration method between both sets.

**Fig 4:**
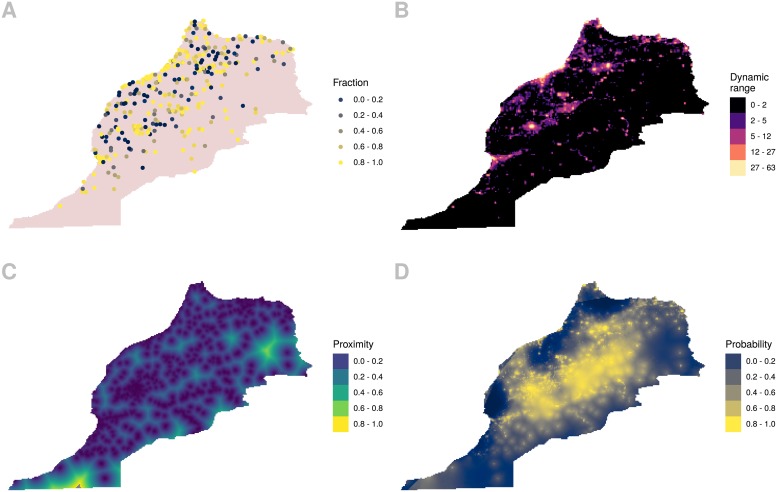
Morocco 2004. A: Fraction of household with electricity access in survey data. B: NTL dynamic range. C: PIA. D: Probability of electricity access.

We are providing coarse estimates of electricity access in a 5 km grid, based on NTL and urbanization approximations. These inputs are able to capture overall trends, but not specific cases. For example, sparsely populated areas with access to electricity would be considered as having low electricity access due to having fewer impervious surfaces and reduced NTL intensity. While our results have a large error (14.3% according to Table 3), the information provided can still be a good guideline where there is no other data source available. For example in the period of study, Morocco only has information of a survey carried on in 2004. Official data from the country reports that it has improved their electricity access from 18% in 1995 to 95% in 2008 [37]. This is in part due to their Rural Electrification Global Program, which has made of Morocco an example of best practices [38]. The official statistics are indeed very useful, but combining survey data from satellite imagery allows us to have a glimpse beyond the administrative level. In Fig 4 we show the survey data, NTL, PIA and the probability of electricity access estimated, all from the year 2004. The survey data do not span all over the country as NTL and LULC do. We can see how the results in panel D are directly associated with the data of panels B and C, and how they provide a continuous representation of the data in panel A.

**Fig 5:**
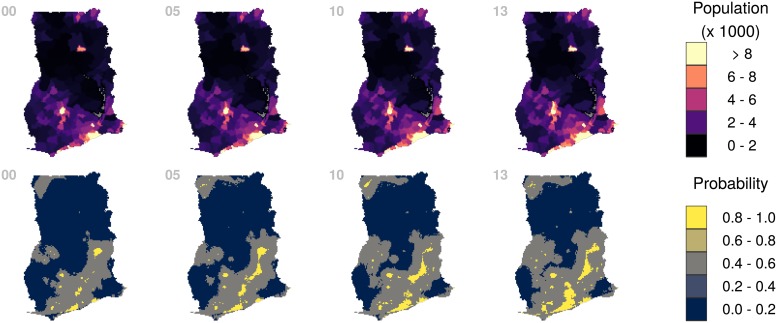
Ghana 2000-2013. From left to right the panels represent the years 2000, 2005, 2010 and 2013. Top panels represent population counts according to GPW data. Bottom panels represent the probability of electricity access.

These datasets could help identifying areas where socioeconomic development has been slower in recent years or they could could help planning aid to underdeveloped regions. For example, Ghana is another country that in recent years has made good progress in electricity access. Like Morocco, this is the result of a national commitment towards increasing population access to energy services and which aims a 100% electrification rate by 2020 [39]. In Fig 5 we show a comparison of population across the country with the probability of electricity access, for the years 2000, 2005, 2010 and 2013. Population data was obtained from the GPW project [32]. The upper panels show where population groups are located, while the lower panels show the areas where there is a higher chance of electricity access. Together, these two series of maps tells us a story of the development of different population groups in the country: electricity access has improved much faster in the south of the country; meanwhile there are large population groups in the north whose progress has been slower.

**Fig 6:**
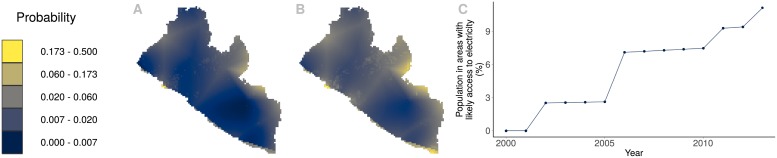
Liberia 2000-2013. A: Probability of electricity access in 2000. B: Probability of electricity access in 2013. C: Population in areas where the probability of electricity access is greater than 0.5.

Historically, Liberia has been among the countries with the lowest household electricity access level. Liberia went through a period of civil war that lasted 14 years and came to an end in 2003. Since then, electrification efforts of the country have included expanding its diesel powered grid and rehabilitating the Mount Coffee hydropower plant [40, 41]. In Fig 6, we show how electricity access changed in this country from 2000 to 2013. Once again, the electricity access maps produced here in combination with the GPW data can help us track this changes.

**Fig 7:**
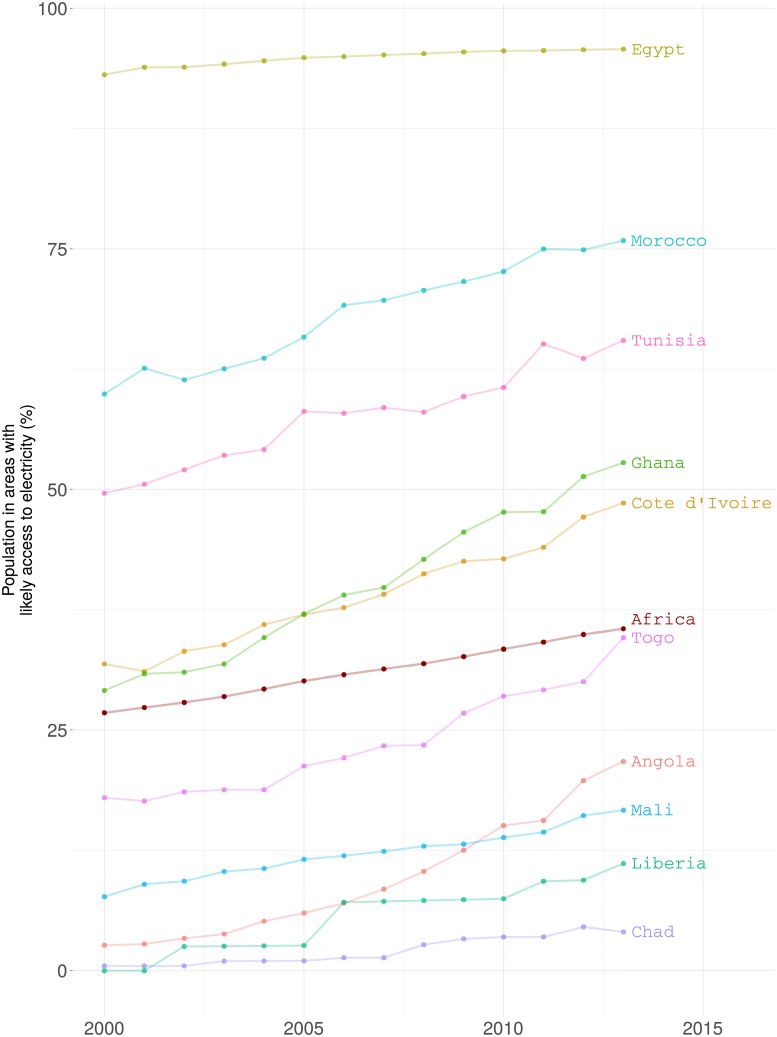
Electrification trends. The bullets represent the electricity access estimates. The overall trend of the 49 countries analyzed is shown in red (only 10 countires are included to make the plot legible).

Having data for the whole continent allows us to make comparisons across countries during different years. As an example, in Fig 7 we show a comparison of electricity access trends between ten different countries as well as the overall trend of the African Continent (we only include 10 countires to make the plot legible). The Figure shows the time series of the percentage of people living in areas where the probability of electricity access is higher than 0.5. We can see how Egypt is well above the Continental average. Conversely, at the bottom of the graph we see how Chad is far behind and with a slow progress. Meanwhile, Togo is progressing fast and getting close to the Continental average. In the year 2000, the African population living in areas likely to have electricity (probability higher than 0.5) was only 26.79%. That number has kept increasing and by the year 2013 it has raised up to 35.52%.

## Conclusion

We have implemented a methodology for interpolating the proportion of households with electricity between 2000 and 2013 across Africa. Our statistical model incorporates information from different sources: survey points per village, annual composites of satellite imagery, land use and land cover.

The aggregation and random displacement of GPS locations as a means of privacy protection represents a source of noise that we cannot remove. In some cases this displacement may imply that the data modeled corresponds in fact to a contiguous grid-cell. Nevertheless, our sensitivity analysis showed that our results are consistent with what could be obtained without displacing the village locations. We followed DHS guidelines that recommend working at a 5 km resolution when using their data.

We included time only as a fixed effect. This may be seen as an unrealistic assumption, as there most be an interaction between space and time: some locations increase their electricity network at different times and rates. However, our model selection methodology discarded adding a spatiotemporal random effect. While there is good spatial coverage of data across the continent, the poor performance of the spatiotemporal model could be due to the fact that data points are very sparse in time. This limits the ability to learn a time effect. In addition, we have to consider that a time component is already implicitly included in the NTL and LULC data.

The information produced here aims to fill in important information gaps regarding electricity access in Africa. We believe that both researchers and planners in this region would benefit from this type of data. Other studies have previously demonstrated a strong association between satellite imagery and different socio-economic indicators, including electricity access or consumption [42–46]. However, to date there exist few continental level data sources at sub-national scale regarding electricity access in the developing world, and sub-Saharan Africa in particular. To our knowledge, this is the first time series of electricity access provided for this period for the whole African continent at this grid-resolution. The 5 km annual grid-maps of the household electricity access and the code used in this project accessible through the links listed in Section A in S1 File.

## Supporting information

S1 File A: Links to Data Sources and Data Outputs. B: Surveys Used. C: Gaussian Process Regression. D: Stochastic Partial Differential Equation and Integrated Nested Laplace Approximation. E: Sensitivity Analysis. F: Model Selection.
(PDF)
