# Supplementary material for: Household electricity access in Africa (2000–2013): Closing information gaps with model-based geostatistics
Source: PLoS One. 2019 May 1;14(5):e0214635. doi: 10.1371/journal.pone.0214635 (PMC6493706; doi:10.1371/journal.pone.0214635)
Supplement: S1 File — A: Links to Data Sources and Data Outputs. B: Surveys Used. C: Gaussian Process Regression. D: Stochastic Partial Differential Equation and Integrated Nested Laplace Approximation. E: Sensitivity Analysis. F: Model Selection. (PDF) [file pone.0214635.s001.pdf]

Supporting Information

A Links to Data Sources and Data Outputs

The information products used here can be accessed through the following links:

- DHS Stat Compiler - `www.statcompiler.com`,
- DHS surveys - `http://dhsprogram.com`,
- NTL - `https://geodata.globalhealthapp.net`,
- LULC - `https://geodata.globalhealthapp.net/lulc/`,
- GPW - `http://sedac.ciesin.columbia.edu`.

The data output as well as the code used in this project can be accessed through the following links:

- Electricity access - `https://geodata.globalhealthapp.net/epaper/`,
- Code - `https://github.com/ric70x7/ElectricityAfrica`.

B Surveys Used

We identified 78 georeferenced surveys (34,198 data points). However not all of the data points had valid coordinates. We considered valid only the GPS coordinates that represented a location inside the country surveyed. After checking for this condition we only kept 69 surveys (30,115 data points). Table B1 in S1 File lists the surveys analyzed and number of data points in each one.

C Gaussian Process Regression

Let $f_{\mathbf{x}} \sim \mathcal{GP}(\mathcal{M}, K)$ be a Gaussian process with domain $\mathcal{X}$, mean function $\mathcal{M}$ and covariance K. The common modeling assumption in GP models is that the response variables $\{y_i\}_{i=1}^n$ are noisy observations of the latent variables $\{f_{\mathbf{x}}\}_{i=1}^n$, and that the noise follows a zero mean Gaussian distribution [1]. Hence

$$y_i \sim \mathcal{N}(f_{\mathbf{x}_i}, \sigma_\epsilon^2), \tag{S1}$$

where σ_ϵ^2 is regarded as noise variance.

In the case of the Geostatistic models implemented in this work, $\mathcal{X}$ represents a 2 dimensional space and $\{\mathbf{x}\}_{i=1}^n$ are a set of spatial indices that vary continuously in the domain $\mathcal{X}$. The spatial process $f_{\mathbf{x}}$ is a random field that follows a multivariate Gaussian distribution with mean $\mathcal{M}$ and covariance K. This methodology is not limited to the assumption of additive Gaussian noise. Other families of distributions can be used, as long as the latent variables are related to the response through some monotonic transformation of the form $\phi : \mathbb{R} \to \mathcal{Y}$, where $\mathcal{Y}$ represents the space of the response variable [2–6].

A binomial noise model, rather than Gaussian, provides an adequate representation of the stochasticity observed in the survey data modeled. Such data correspond to the number of households with access to electricity out of a total number of households in a village. We can assume an inverse logit transformation that shrinks the real space into

B1 Table. Survey points used. For each country-year we present above in square brackets the Survey ID, below at the left the total number of data points, and below at the right the number of data points considered valid and used. The country names are abbreviated according their ISO 3166-1 alpha-3 code as defined in Table B2 in S1 File.

Country	2000	2001	2002	2003	2004	2005	2006	2007	2008	2009	2010	2011	2012	2013
AGO								[282]<br>115/115				[395]<br>238/227		
BDI											[346]<br>376/376		[446]<br>200/200	
BEN		[211]<br>247/247					[289]<br>750/0						[420]<br>750/746	
BFA				[230]<br>400/397							[329]<br>573/541			
CIV													[311]<br>351/341	
CMR					[232]<br>466/464							[337]<br>578/577		
COD								[239]<br>300/293						
EGY	[145]<br>1000/977			[240]<br>980/918		[272]<br>1359/1285			[294]<br>1267/1231					
ETH	[147]<br>539/535					[248]<br>535/528						[359]<br>596/571		
GAB	[148]<br>249/0												[402]<br>336/329	
GHA				[235]<br>412/406					[301]<br>411/403					
GIN						[249]<br>295/285							[391]<br>300/300	
KEN				[216]<br>400/394						[300]<br>398/394				
LBR								[271]<br>298/268		[330]<br>150/147		[361]<br>150/147		[435]<br>322/316
LSO					[256]<br>405/381					[317]<br>400/395				
MAR					[214]<br>480/476									
MDG					[207]<br>300/0					[296]<br>594/581		[396]<br>267/265		[456]<br>274/273
MLI		[159]<br>402/399					[276]<br>407/405							[405]<br>413/413
MOZ				[174]<br>604/0								[362]<br>610/609		
MWI	[158]<br>560/560				[251]<br>521/520						[333]<br>849/827		[432]<br>140/140	
NAM	[205]<br>260/257							[284]<br>500/491						[363]<br>550/548
NGA				[223]<br>362/360					[302]<br>886/885		[392]<br>239/238			[438]<br>896/889
RWA	[165]<br>443/0					[252]<br>462/456			[323]<br>249/246		[364]<br>492/492			[449]<br>159/0
SEN						[233]<br>376/363	[293]<br>150/0			[338]<br>320/318		[365]<br>391/385		[423]<br>200/200
SLE									[324]<br>353/350					[450]<br>435/430
SWZ								[259]<br>275/270						
TZA						[253]<br>475/0					[345]<br>475/454			
UGA		[169]<br>298/267					[266]<br>368/336			[332]<br>170/170		[399]<br>404/400		
ZMB			[206]<br>320/0					[278]<br>319/319						
ZWE							[260]<br>398/396					[367]<br>406/393		

the interval $\mathcal{Y} = (0, 1)$. The model of the households number with electricity per village, if no fixed effects are considered, is then given by

$$y_i \sim \text{Binomial}(\phi(f_{\mathbf{x}_i}), n_i), \tag{S2}$$

where n_i represents the total number of households associated to the i-th village.

D Stochastic Partial Differential Equation and Integrated Nested Laplace Approximation

Spatial models commonly rely on covariance functions that depend on the Euclidean distance $\|\mathbf{x}_i - \mathbf{x}_j\|$. The Matérn covariance, for example, is defined as

$$K(\mathbf{x}_i, \mathbf{x}_j) = \sigma^2 \frac{2^{1-\nu}}{\Gamma(\nu)} \left(\frac{\sqrt{2\nu}\|\mathbf{x}_i - \mathbf{x}_j\|}{\ell} \right)^{\nu} K_\nu \left(\frac{\sqrt{2\nu}\|\mathbf{x}_i - \mathbf{x}_j\|}{\ell} \right), \tag{S3}$$

B2 Table. ISO 3166-1 alpha-3 codes. This list only contains countries from which we analyzed georeferenced survey data as described in Table B1 in S1 File.

Code	Country	Code	Country
AGO	Angola	MAR	Morocco
BDI	Burundi	MDG	Madagascar
BEN	Benin	MLI	Mali
BFA	Burkina Faso	MOZ	Mozambique
CIV	Cote d'Ivoire	MWI	Malawi
CMR	Cameroon	NAM	Namibia
COD	Congo Democratic Republic	NGA	Nigeria
EGY	Egypt	RWA	Rwanda
ETH	Ethiopia	SEN	Senegal
GAB	Gabon	SLE	Sierra Leone
GHA	Ghana	SWZ	Swaziland
GIN	Guinea	TZA	Tanzania
KEN	Kenya	UGA	Uganda
LBR	Liberia	ZMB	Zambia
LSO	Lesotho	ZWE	Zimbabwe

where ℓ and ν are non-negative parameters, and K_ν is the modified Bessel function of second kind and order ν.

Exact inference with a GP regression has a computational complexity of $\mathcal{O}(n^3)$, where n is the number of training cases. Such a cost arises from the need to invert the covariance matrix. Thus, large datasets prevent the use of $\mathcal{O}(n^3)$ algorithms. An alternative is provided by the stochastic partial differential equation (SPDE) approach [7]. The SPDE approach approximates the Gaussian random field (assuming a Matérn covariance) with a Gaussian Markov random field allowing the use of a sparse precision matrix and reducing the complexity to $\mathcal{O}(n^{\frac{3}{2}})$.

The SPDE approach can be applied for both stationary and non-stationary processes. In the latter case, used in this work, the starting point of the approximation is the equation

$$(\kappa(\mathbf{x})^2 - \Delta)^{\alpha/2}(\tau(\mathbf{x})f_{\mathbf{x}}) = w_{\mathbf{x}}, \tag{S4}$$

where $\kappa(\mathbf{x})$ and $\tau(\mathbf{x})$ are parameters that vary in space, Δ is the Laplacian, α controls the smoothness (here we assumed $\alpha = 2$, which is the default value in R-INLA), and $w_{\mathbf{x}}$ is a Gaussian spatial white noise process.

The nominal approximation to the variance and range of the spatial correlation are given by

$$\sigma_n^2(\mathbf{x}) \approx \frac{1}{4\pi\kappa(\mathbf{x})^2\tau(\mathbf{x})^2}, \tag{S5}$$

$$r_n(\mathbf{x}) \approx \frac{\sqrt{8}}{\kappa(\mathbf{x})}. \tag{S6}$$

The range is defined as the distance at which the spatial correlation is close to 0.1

The SPDE representation also makes it possible to use the Integrated Nested Laplace Approximation to handle the non-linear transformation on the latent variable [8]. The INLA model computes the posterior marginals of the latent field through a step-wise implementation of a simplified Laplace approximation with a cost of $\mathcal{O}(n^2 \log n)$. We used the SPDE and INLA algorithms provided by the R-INLA package [9, 10].

E Sensitivity Analysis

Statistical inference is affected by the random displacement of cluster locations. To mitigate this effect DHS has proposed some guidelines when using their data along with predictors or covariates defined spatially [11]. These guidelines cover three specific cases: distance-base analyses, raster-based analyses and point-in-polygon analyses. The model we propose falls in the category of raster-based as this is the format in which the covariate information we are using is stored: a regular grid of values that represent a pixel area. In this case, DHS guidelines suggest that covariates are not computed using direct cell extraction (pixel value that corresponds to the GPS location), but using an average value across a 5 km buffer around the GPS location reported.

We performed a simulation analysis to assess the impact of the locations displacement. We generated a synthetic dataset of 10,000 observations according to the pseudo-code shown in Table E1 in S1 File. First, we fitted a GLM to a sample of the survey data and assumed that the residuals were given by a spatial random field. Second, we used these residuals and the GLM fitted values as the inverse logit transformation of the mean of a Binomial random process.

Being this merely a spatial phenomenon, we did not considered time in this section of the analysis. The synthetic dataset was based on a sample of the available survey data between 2008 and 2012. We sampled across multiple years to ensure a larger spatial coverage of the data, but constrained to a window of four years to reduce variations due to time.

E1 Table. Synthetic data generation. Steps followed to generate the synthetic dataset for sensitivity analysis.

Pseudo code for data simulation	
Sample m observations $\{y_i, n_i\}$ from data.	$\#y_i$: positives; n_i: total
For each observation $i \leftarrow 1$ to m:	
For each covariate $j \leftarrow 1$ to q:	$\#q$: number of covariates
Compute z_{ij} using a 5 km buffer	$\#z_{ij}$: covariate j at point i
Fit a GLM and estimate $\{\hat{\beta}_j\}$.	$\#\beta_j$: regression parameter j
Compute the errors vector $\hat{\boldsymbol{\varepsilon}} = [{}^{y_i}/_{n_i} - {}^{\hat{y}_i}/_{n_i}]$.	
For each observation $i \leftarrow 1$ to m :	
Compute $\eta_i = \sum \hat{\beta}_j z_i^{(j)} + \hat{\varepsilon}_i$	
Compute $p_i = {}^1/_{1+\exp(-\eta_i)}$	
Simulate $\tilde{y}_i \sim \text{Binomial}(p_i, n_i)$	

For the purposes of this exercise we assumed that the synthetic data locations, which correspond to the survey locations reported, were not displaced. We then generated an new obfuscated dataset (with displaced locations) based on these data, following the pseudo-code of Table E2 in S1 File. DHS methodology for data protection displaces cluster locations up to 2 km in urban areas and up to 5 km in rural areas, with 1% of the locations displaced up to 10 km. Our algorithm did not displaced the locations using distances measured in the metric system, but in coordinates degrees. We assumed that 1 degree is equivalent to 111 km, which is the approximate proportion of degrees to kilometers in the Equator. That means that the offset we used in this simulation is larger the farther from the Equator. In addition, for simplicity we did not make any distinction between urban and rural clusters and made a displacement up to 5/111 degrees for 99% of the data points and up to 10/111 for the remaining 1%. Fig. E1 in S1 File shows the displacement of a sample of points, with respect to their *assumed* original location, and a histogram of the displacement magnitudes applied to the whole dataset. Note that the proportion of points tend to increase as the offset increases because the area of the discs around the original location is larger the farther we are from it.

E2 Table. Locations displacement. Steps followed to obfuscate the GPS locations of the synthetic dataset according to DHS methodology.

Pseudo code for locations displacement	
For each point $i \leftarrow 1$ to m:	
Simulate $u \sim \text{Uniform}(0, 1)$	
If $u < 0.99$:	
Define radius $r = {}^{5}/_{111}$:	
Else:	
Define radius $r = {}^{10}/_{111}$:	
Define buffer of radius r around $\mathbf{x}_i$	$\mathbf{x}_i$: GPS coordinates of point i
Randomly choose new $\mathbf{x}_i^* \in B(\mathbf{x}_i)$	

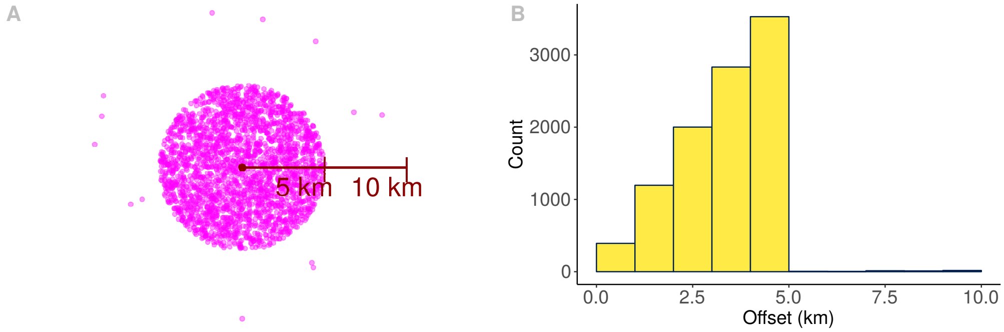

E1 Fig. Coordinates displacement. A: Offset with respect to the *assumed* original location (only showing 25% of data). B: Histogram of the offsets magnitude. Distances are computed assuming 1 degree = 111 km.

We fitted a Geostatistic model to both datasets, non-obfuscated and obfuscated, according to

$$y_i \sim \text{Binomial}\left(\phi(\eta_i), n_i\right) \tag{S7}$$

and

$$\eta_i = \sum_{i=1}^{7} \tau_i + \sum_{i=1}^{3} \beta_i z_{[\mathbf{x}_i]} + f_{\mathbf{x}_i}, \tag{S8}$$

where ϕ is the logit transformation, τ_i are coefficients of the binary variables indicating the LULC category, z_i are the continuous covariates: IAP, NTL and PIA; β_i are the coefficients of the continuous covariates; $f_{\mathbf{x}_i}$ is the spatial random field and $\mathbf{x}_i$ are the spatial reference points.

Fig. E2 in File S1 shows a comparison of the marginal distribution of the fixed effects parameters when using obfuscated and non-obfuscated datasets. Also the *true* parameters are shown with a vertical red line. In general, the true parameters are not covered by areas of high density of the marginal distributions, which reflects that both cases are missing the right value. The distributions of all parameters with both datasets present a very similar dispersion. In addition, in 6 of the 10 parameters estimated, there is a clear overlap between the marginal distributions of the two models. This suggests that results tend to be consistent and not highly affected by the locations displacement.

Fig. E3 in File S1 shows a comparison of the marginal distribution of the spatial hyperparameters when using obfuscated and non-obfuscated datasets. In this case we do not have a ground truth to compare with. The marginal distribution of κ is shifted to the right when using obfuscated data. We will not dwell on the comparison of marginal distributions of this parameter since its interpretation is not intuitive. We will expand more on the nominal variance and the nominal range which depend on κ and

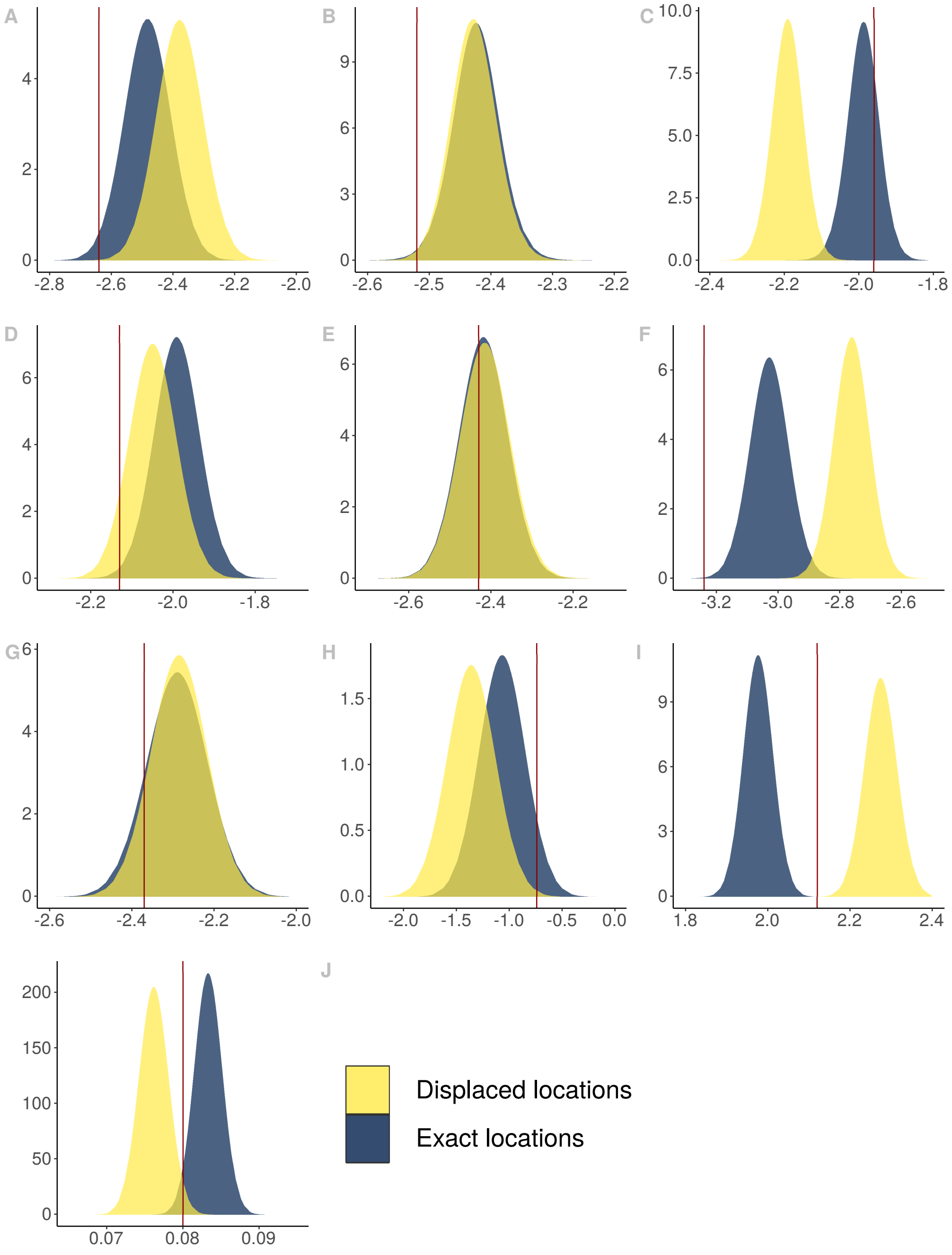

E2 Fig. Marginal distribution of the fixed effects parameters. A: Impervious surface. B: Low biomass. C: High biomass. D: Bare soil. E: Sand. F: Rock. G: Water. H: IAP. I: PIA. J: NTL. Sensitivity to obfuscation of fixed effects. The values of the parameters used to generate the synthetic data are indicated with a red line.

are more easy to interpret. The nominal variance of the model with obfuscated data is larger, which means a higher variability of the spatial random field and is possibly driven by the noise due to the locations displacement. The nominal range of the spatial covariance is smaller when modeling obfuscated data. This means that the radius at

which two points present a correlation above 0.1 is shorter when data has been displaced. This reduction in the range prevents correlating points that would be assumed to be correlated if using the range of the non-obfuscated data. As a result, the model for obfuscated data relies less on the proximity of observations. The last seems adequate considering that we are not certain about the true location of the data. Once again, this lack of certainty may be a driver of the larger nominal variance.

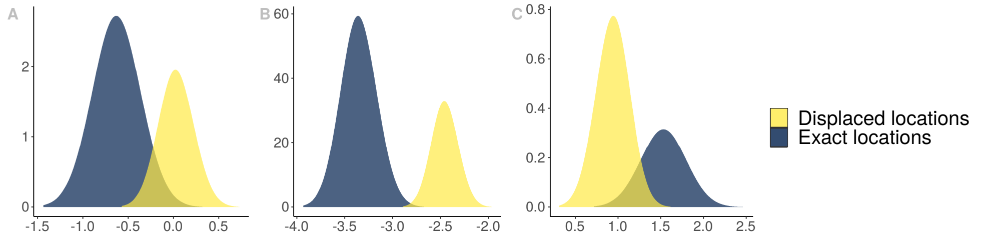

E3 Fig. Marginal distribution of the hyperparameters. A: Hyperparameter κ. B: Nominal variance σ_n^2. C: Nominal range r_n. The support of the distributions is shown in log-scale.

Fig. E4 in File S1 shows a diagram explaining the effect of the reduction in the range. The red points represent the actual locations of three observations. The radius of the blue circle represents a hypothetical *large* nominal range. All points within the blue circle are correlated with A ($\rho > 0.1$ to be more precise). Such is the case of point C, but not of point B. The magenta dots represent potential displaced locations of points B and C. For the sake of this example we do not displace point A. If we use the *large* nominal range, point A will be still correlated with most of the displaced locations of C. In addition, A will also be assumed to be correlated with some displaced locations of B. However, if instead we use a *short* nominal range (radius of the yellow circle), A will be considered to be correlated only to a few displaced locations of C, and it will no longer be correlated with any of the displaced locations in B.

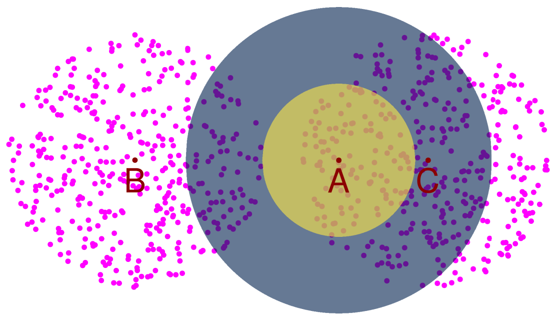

E4 Fig. Nominal range and location displacement. Diagram of the effect of the nominal range reduction on obfuscated data. The red points A, B and C represent non-displaced data. The magenta points represent possible displaced locations of points B and C. The circles represent areas where the correlation with point A is above 0.1, according to the nominal range (circle radius) of each one.

In addition to the comparison of the marginal distributions, we compared the odds ratio of the fixed effects. First, we compared the true values vs the estimates with obfuscated data (see Table E3 in S1 File); second, we compared the estimates using non-obfuscated data vs obfuscated data (see Table E4 in S1 File). In both tables we see that odds ratios are consistent. Only NTL and PIA present ratios larger than one. The

rest of odds ratios are smaller than one. Moreover, the difference between the ratios of obfuscated data estimates and the base of comparison (true values or non-obfuscated estimates) is in general small relative to the magnitude of such base estimates. These tables show that the random displacement is not perturbing too much the fixed effects estimates.

E3 Table. Odds ratio estimates (true vs displaced). Comparison of the odds ratios used to generate the synthetic data and their mean estimates using displaced locations.

Variable	True value	Estimate (obfuscated)	Difference
Impervious surface	0.07	0.09	0.02
Low biomass	0.08	0.09	0.01
High biomass	0.14	0.11	0.03
Bare soil	0.12	0.13	0.01
Sand	0.09	0.09	0.00
Rock	0.04	0.06	0.02
Water	0.09	0.10	0.01
IAP	0.48	0.26	0.22
PIA	8.33	9.72	1.38
NTL	1.08	1.08	0.00

E4 Table. Odds ratio estimates (exact vs displaced). Comparison of the odds ratio mean estimates when using exact and displaced locations.

Variable	Estimate (exact)	Estimate (obfuscated)	Difference
Impervious surface	0.08	0.09	0.01
Low biomass	0.09	0.09	0.00
High biomass	0.14	0.11	0.03
Bare soil	0.14	0.13	0.01
Sand	0.09	0.09	0.00
Rock	0.05	0.06	0.01
Water	0.10	0.10	0.00
IAP	0.34	0.26	0.09
PIA	7.22	9.72	2.50
NTL	1.09	1.08	0.01

Finally, in Fig. E5 in File S1 we compare the target values estimates: in panel A, we compare the estimates using the non-obfuscated data vs the true target values; in Panel B, we compare the estimates using the obfuscated data vs the true values; in Panel C, we compare the estimates using the obfuscated data vs the estimates using the non-obfuscated data. By target values we mean the inverse logit of the probability of having electricity access. This is the η_i value on the left hand side of Eq S8. Panels A and B show that the target estimates are aligned around the true values, and panle C shows that the estimates obtained using exact and displaced locations are consistent between them. Another way of viewing this is that the target value estimates with displaced locations are an approximation of the estimates that we could obtain with the exact locations. As expected, the estimates when using obfuscated data are more spread than when using non-obfuscated data. However the difference is not too large. In fact, the sum of squared errors (SSE) for the data in panel A is 624.54, while the SSE for the data in panel B is 659.01. Thus, using obfuscated data increases the SSE only a 5.52%.

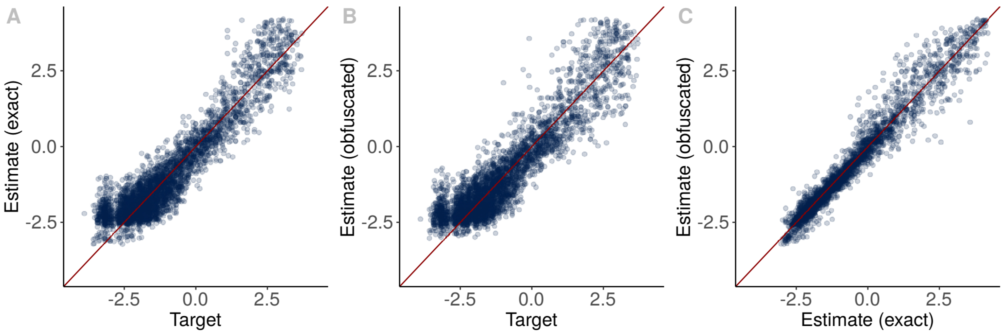

E5 Fig. Sensitivity to obfuscation of target estimates. A: Estimates with exact locations vs target values, B: Estimates with obfuscated locations vs target values, C: Estimates with obfuscated locations vs Estimates with The target values are defined as the logit transformation of the probability estimates of electricity access. The plot only shows points for target values different from 0 or 1, since these values are not defined for the logit transformation.

F Model Selection

We compared 4 different models. Model 1 is just a GLM with all the variation explained by the covariates of land cover type, IAP, PIA and NTL. Model 2 is a Geostatistic model that extends Model 1 by adding a spatial random field. Model 3 extends Model 2 by adding the year of observation as a covariate. Model 4 extends Model 2 by incorporating an autoregressive component to the random field across time.

The performance of these models was compared using the conditional predictive ordinate (CPO) as criterion. CPO is defined as the probability of observing value y_i given the rest of the values. This is expressed as

$$\mathrm{CPO}_i = p(y_i|\mathbf{y}_{-i}), \tag{S9}$$

where $\mathbf{y}_{-i} = \{y_j\}_{j \neq i}$. Note that CPO is computed using leave-one-out cross-validation. For this part of the analysis we used only 21,080 observations, that correspond to 70% of the survey points with displaced locations. Table F1 in S1 File shows a summary of the models comparison. The model with largest sum of CPO is Model 3, and therefore it this was the one used for estimating the probability of electricity access. Model 3 can be expressed as

$$y_i \sim \mathrm{Binomial}(\phi(\eta_i), n_i) \tag{S10}$$

and

$$\eta_i = \sum_{i=1}^{7} \tau_i + \sum_{i=1}^{4} \beta_i z_{[\mathbf{x}_i, t_i]} + f_{\mathbf{x}_i}, \tag{S11}$$

where ϕ is the logit transformation, τ_i are the fixed effect estimates LULC categories, z_i are the remaining covariates (IAP, PIA, NTL and time), β_i are the regression parameters of such covariates, $f_{\mathbf{x}_i}$ is the spatial random field, and $\mathbf{x}_i$, t_i are the space and time reference points.

NTL and PIA have odds ratios larger than one, which reflects a positive association with the probability of electricity access. The difference in the magnitude of the ratios is due to the scale of the variables: NTL dynamic range goes from 0 to 63, while PIA values are constrained between 0 and 1. On the other hand, the category impervious surface and the variable IAP have odds ratios smaller than one, which describes a negative association. This is counterintuitive, as we expect urbanization processes to be positively associated with electricity access. However, the variable that dominates this relationship is PIA, which is positive.

F1 Table. Models comparison. Parameter estimates and CPO values computed for the different models implemented.

	Model 1	Model 2	Model 3	Model 4
Fixed effects (odds ratio)				
Impervious surface	0.09	0.10	0.07	0.07
Low biomass	0.07	0.08	0.05	0.06
High biomass	0.10	0.06	0.04	0.05
Bare soil	0.11	0.16	0.11	0.13
Sand	0.07	0.07	0.05	0.05
Rock	0.08	0.08	0.05	0.06
Water	0.07	0.07	0.05	0.05
IAP	0.48	0.14	0.11	0.12
PIA	10.46	8.74	8.18	12.98
NTL	1.08	1.08	1.09	1.08
Year	-	-	1.05	-
Hyperparameters				
$\log \kappa$	-	1.43	0.94	0.93
$\log \sigma_n^2$	-	2.71	2.71	3.13
$\log r_n$	-	-0.39	0.10	0.11
ρ	-	-	-	0.62
Selection criterion				
$\sum_i \log \mathrm{CPO}_i$	-133×10^3	-120×10^3	-119×10^3	-159×10^3

G Model Validation

Once the model was trained, we predicted the probability of a household having electricity at each of the locations in V1. Then we classified the locations as with electricity or without electricity. The datasets used contain information about the proportion of households with electricity within the cluster (see Table 1), as opposed to a binary classification. We simulated binary classes according to this proportion following the pseudo-code of Table G1 in S1 File.

G1 Table. Synthetic classes. Steps followed to simulate binary classes for set V1.

Pseudo code for random class generation	
C = [ ]	
For each point $i \leftarrow 1$ to $\|V1\|$:	
For j in 1 to 100:	#100 classes per observation
Simulate $c \sim$ Bernoulli(y_i/n_i)	
C.append(c)	

Using the synthetic classes of V1 we computed the accuracy, precision, sensitivity and confusion matrices of our predictions. Results are shown in Tables 2 and 3.

References

1. Williams CKI, Rasmussen CE. Gaussian processes for Machine Learning. MIT Press; 2006.

2. Neal RM. Regression and classification using Gaussian process priors. Bayesian Statistics. 1998;6:475–502.

3. Williams CKI, Barber D. Bayesian classification with Gaussian processes. Transactions on Pattern Analysis and Machine Intelligence. 1998;20(12):1342–1351.

4. Adams RP, Murray I, MacKay DJC. Tractable nonparametric Bayesian inference in Poisson processes with Gaussian process intensities. In: Bottou L, Littman M, editors. Proceedings of the International Conference in Machine Learning. vol. 26. ACM. San Francisco, CA: Morgan Kaufmann; 2009. p. 9–16.

5. Minka T. Expectation Propagation for approximate Bayesian inference. In: Breese JS, Koller D, editors. Uncertainty in Artificial Intelligence. vol. 17. San Francisco, CA: Morgan Kaufmann; 2001. p. 362–369.

6. Opper M, Winther O. Gaussian Processes for Classification: Mean Field Algorithms. Neural Computation. 2000;12:2655–2684.

7. Lindgren F, Rue H, Lindström J. An explicit link between Gaussian fields and Gaussian Markov random fields: the stochastic partial differential equation approach. Journal of the Royal Statistical Society: Series B (Statistical Methodology). 2011;73(4):423–498.

8. Rue H, Martino S, Chopin N. Approximate Bayesian inference for latent Gaussian models by using integrated nested Laplace approximations. Journal of the Royal Statistical Society: Series B (Statistical Methodology). 2009;71(2):319–392.

9. Blangiardo M, Cameletti M. Spatial and spatio-temporal bayesian models with R-INLA. John Wiley & Sons; 2015.

10. Martins TG, Simpson D, Lindgren F, Rue H. Bayesian computing with INLA: new features. Computational Statistics & Data Analysis. 2013;67:68–83.

11. Perez-Heydrich C, Warren JL, Burgert CR, Emch M. Guidelines on the use of DHS GPS data; 2013.
